# Comparison of MRI and endoscope ultrasound detection in preoperative T/N staging of gastric cancer

**DOI:** 10.3892/mco.2013.103

**Published:** 2013-04-12

**Authors:** CUI LEI, LIMING HUANG, YANLIN WANG, YILING HUANG, YURONG HUANG

**Affiliations:** 1 Department of General Surgery, Yichang Central People’s Hospital, China Three Gorges University, Yichang, Hubei 443000;; 2 Institute of Molecular Biology of China Three Gorges University, Yichang, Hubei 443000;; 3 Department of Oncology, Zhongnan Hospital, Wuhan University, Wuhan, Hubei 430071, P.R. China

**Keywords:** gastric cancer, endoscopic ultrasound, magnetic response imaging, preoperative clinical staging

## Abstract

Gastric cancer is a common malignancy and cause of mortality. The aim of this study was to evaluate the accuracy of magnetic resonance imaging (MRI) and endoscopic ultrasound (EUS) detection in preoperative clinical T/N staging in gastric cancer. Thirty-eight patients diagnosed with gastric cancer by gastroscopy and pathological examination were included in the study. All 38 patients underwent MRI and EUS detection prior to surgery. The accuracy of MRI, EUS and MRI+EUS was evaluated according to postoperative pathological staging. Results identified the accuracy of EUS, MRI and EUS+MRI in T clinical staging to be 86.64, 73.68 and 89.47%, respectively (MRI vs. EUS+MRI, P=0.035), while the accuracy for N clinical staging was 65.78, 68.42 and 71.05%, respectively (P>0.05). The accuracy rate in EUS and EUS+MRI detection in N0 stage was markedly higher compared with that in MRI (100 vs. 86.67%, P=0.032), whereas the rate in EUS detection in N2 stage was lower compared with that in MRI and EUS+MRI (45.45 vs. 54.54%, P=0.021). Thus, both MRI and EUS had a higher accuracy in preoperative T/N staging. Additionally, the accuracy rate was improved significantly when the two procedures were combined.

## Introduction

Gastric cancer, a common malignant tumor in China, has the fourth highest incidence, and is the second highest cause of cancer death worldwide (1). Due to the non-specific symptoms in the early stages of the disease, the majority of patients present with advanced gastric carcinoma at the initial diagnosis, resulting in a poor prognosis. Preoperative staging is crucial for individual treatment and evaluation of prognosis. Magnetic resonance imaging (MRI) and endoscopic ultrasound (EUS) have been widely used to detect the invasion depth and lymph node metastasis (2). The aim of this study was to evaluate the accuracy of MRI, EUS and EUS+MRI in T/N staging in gastric cancer.

## Patients and methods

### Patients

Fifty-two patients were initially diagnosed with gastric cancer by gastroscopy and pathological examination between October, 2010 and December, 2011. Following the exclusion of 14 patients due to neoadjuvant chemotherapy or palliative treatment, 38 patients (26 males and 12 females; average age, 52 years; range, 31–82 years) were included in the study. Moderately differentiated adenocarcinoma was identified in 13 patients, poorly differentiated adenocarcinoma in 18, signet-ring carcinoma in 5 and mucinous adenocarcinoma in 2 patients. Each patient underwent MRI and EUS for tumor detection one week prior to surgery. The results were assessed by professional doctors.

### EUS detection

Detection of tumors was carried out using Olympus Ultrasonic diagnostic apparatus (GF-UMQ130, Tokyo, Japan) and a pre-endoscopic ultrasound probe, at a frequency of 7.5 MHz. Patients were required to fast for 20 min prior to the procedure and were then administered 10 mg of 654-2 intramuscularly. The patient was placed in a left lateral position and endoscopic ultrasound was performed subsequent to removal of air and water injection (350 ml) to fill the bladder. The stomach was identified in the region from duodenum to cardia using the transducer.

### MRI detection

1.5T superconductive magnetic resonance imaging (1.5T Signa HD; GE Healthcare, Pittsburgh, PA, USA) was applied to all 38 patients. The 1.5T superconducting magnetic resonance scanner imaging coil, which has an 8-channel body coil scan level, was used to obtain cross-sectional and coronal oblique crown surface images, as required. Images were scanned according to the sequence: T1 FSPGR (TR/TE=180/3.3), T2 SSFSE (TR/TE=1800/79.5), T2 ASSET (TR/TE=6667/85.8) and diffuse-weighted images (DWI) (TR/TE=1000/74.9, b=600 sec/mm^2^). LAVA dynamic contrast-enhanced scan with fat suppression (TR/TE=4.2/2.0) at 5 mm and with a layer distance of 1 mm was performed. Images were set to a field of view (FOV) of 38×38, and matrix of 244×256. The region from the cardia to the entire stomach was scanned. Magnetic resonance contrast agent gadolinium acid Portuguese amine (Gd-DTPA; 20 ml) was injected via cubital vein injection. Dynamic contrast-enhanced scan time was 18 sec, with the total of the three dynamic contrast-enhanced scans lasting ∼4 min. Patients were required to fast for 20 min prior to administration of the test. A total of 10 mg of 654-2 was injected intramuscularly, prior to ingestion of 1,000 ml warm water. The patient was placed in a supine position and MRI examination was performed.

### Surgery and pathological examination

Following surgery, the resected specimens and perigastric lymph nodes were classified according to the Japanese Gastric Cancer Society. Over 15 lymph nodes were resected for pathological examination as is the general requirement, and all the specimens obtained were assessed by a specialized pathologist.

### Criteria

Criteria for gastric staging were determined according to TNM staging (3). Evaluation for MRI T staging criteria were based on the depth of invasion and were as follows (4): T1, obvious lesions were not detected, nor was the basic integrity of the submucosa disrupted; T2, full thickness tumor infiltration of the stomach, although the outer boundary remained smooth or the slightly enhanced outer layer was still intact; T3, full thickness tumor infiltration of the stomach, with an irregular outer boundary or in a grid pattern or mild enhancement of the outer layer destruction; T4, tumor infiltration of the surrounding tissues and organs. A normal gastric wall comprises a five-layer structure; thus, tumors were classified depending on whether they exhibited this normal five-layer structure of the gastric wall thickening or whether they exhibited hypoechoic mass destruction in the EUS. The degree of tumor infiltration was determined by EUS with the undetermined level (5).

The American Joint Committee on Cancer (AJCC, 7th edition) of TNM staging was used for N staging of regional lymph nodes of gastric cancer (3). When the short diameter of perigastric and distalis perigastric lymph nodes was >5 and >6 mm, respectively, they were considered metastatic lymph nodes. Perigastric lymph nodes were divided into groups 1–6, including the left cardia, right side of the lesser curvature of the stomach, the gastric antrum, and the regions above and below the pyloric lymph nodes, as well as the remaining peri-gastric lymph nodes (groups 7–16). EUS was used to identify clear boundary hypoechoic metastatic lymph nodes, while non-metastatic hyperechoic lymph nodes exhibited fuzzy boundaries.

### Statistical analysis

The results were assessed by the McNemar test. P-values were two-sided, and P<0.05 was considered to indicate a statistically significant difference. Statistical analysis was performed using SPSS 19.0 software.

## Results

### Comparison of T staging

According to the AJCC 7th edition, staging criteria, surgical and pathological findings were: pT1 26.31% (10/38), pT2 21.05% (8/38), pT3 39.41% (15/38), pT4 13.15% (5/38). Compared with the surgical and pathological staging, the accuracy of EUS, MRI and EUS+MRI examination in T staging were 86.84, 73.68 and 89.47%, respectively. EUS and EUS+MRI were statistically significant compared with MRI (EUS vs. MRI, P=0.04, MRI vs. EUS+MRI, P=0.035) (Table I).

In T1 gastric cancer, results demonstrated EUS, MRI and EUS+MRI accuracy to be 90, 70 and 90%, and EUS and EUS+MRI were statistically significant compared with MRI (P=0.032). In T2 stage gastric cancer, results demonstrated the EUS, MRI and EUS+MRI accuracy to be 77.78, 66.67 and 90.00%, and the difference between EUS+MRI and EUS/MRI was statistically significant (EUS+MRI vs. EUS, P=0.04, EUS+MRI vs. MRI, P=0.002). The accuracy of EUS+MRI was significantly higher than that of MRI or EUS alone.

### Comparison of N staging

According to the AJCC 7th edition, staging, surgical and pathological findings were: PN0 39.47% (15/38), pN1 23.68% (9/38), pN2 28.94% (11/38), pN3 7.9% (3/38). Compared with the surgical and pathological staging, the accuracy rates for EUS, MRI and EUS+MRI tumor detection were 65.78, 68.42 and 71.05%, respectively. No significant differences were observed for the three groups (Table II). The N0 detection accuracy rates in EUS and EUS+MRI detection were significantly higher those for MRI (100 vs. 86.67%, P=0.032), while results for EUS demonstrated a lower accuracy rate compared with MRI and EUS+MRI for N2 staging detection (45.45 vs. 54.54%, P=0.021). MRI assessment in N3 showed greater sensitivity compared with EUS. Accuracy was achieved in 3 of 4 cases in N3 staging patients using MRI, while accuracy was achieved in only 1 case with EUS detection. Due to the small sample size, the latter sample was not included for statistical analysis.

## Discussion

Endoscopic therapy, surgery, chemotherapy and radiotherapy are prevalent in the treatment of gastric cancer. When diagnosed in the early stages of gastric cancer, patients had a good prognosis following surgical treatment. Endoscopic treatment (endoscopic mucosal resection or endoscopic submucosal dissection) is a viable option and has fewer side-effects in mucosal and submucosal carcinoma. However, in advanced gastric cancer, treament including laparotomy, radiotherapy, chemotherapy and palliative supportive care exhibit severe side-effects with poor prognosis. Accurate preoperative staging is crucial for clinical treatment programs, the determination of the surgical approach, as well as significant prognostic assessment. For already confirmed infiltration of surrounding organs or transferred cases, improved treatment options should be considered in order to maximize benefits for the patients, and reduce the risk of surgery. EUS+MRI clearly show the organization planning for each level of the stomach wall and adjacent organs. Thus, the two methods have a high accuracy rate in preoperative T/N staging.

Ganpathi *et al* (6) and Tsendsuren *et al* (7) reported that the overall accuracy of EUS in preoperative T staging in gastric cancer patients was 63.0–88.0%. Findings of this study have shown the overall accuracy to be 86.84%, consistent with previous studies (6,7). EUS is a reliable detection method with a high accuracy rate in preoperative staging in gastric cancer. A meta-analysis (8) assessing the diagnostic accuracy of EUS preoperative TN staging analysis was conducted, the results of which showed that EUS exhibits sensitivity in preoperative T staging, with accuracy of T1-T4 being 84.0, 78.8, 78.3 and 80.4%, respectively, while the specificity of T1-T4 was 97.0, 90.5, 88.2 and 95.3%, respectively. Of note is the highest diagnostic accuracy of EUS for T1 gastric cancer.

Large differences have been reported in the literature with regard to preoperative T staging in MRI detection. The accuracy of MRI in preoperative T staging is 73–88%, with an accuracy rate in T1–T4 of 75-100, 63–80, 78.6–96 and 40–100%, respectively. In this study, the accuracy of MRI in T staging was 73.68%, with an accuracy rate in T1–T4 of 70, 66.67, 73.77 and 80%, respectively, which is similar to the accuracy rate reported in the literature. Tumor tissue was identified as moderate or low signal in the multi-scan sequence, and the high signal and favored lesion location was shown in DWI, although this did not improve the accuracy of the T stage. Local thickening and abnormal enhancement was evident in LAVA dynamic enhancement. The performance of advanced gastric cancer in the dynamic contrast-enhanced scan was gradually enhanced from mucosa to serosa, thus it improved the detection rate and increased the accuracy of the T staging (9). By comparing the differentiation of the enhancement between tumor site and adjacent organs, we can determine whether the adjacent organ was infiltrated. Additionally, LAVA dynamic enhanced scanning with fat suppression technique is an improved method for distinguishing between T3 and T4 staging. This finding is crucial for T3/T4 identification ability of gastric cancer patients treated with surgery.

Findings of this study demonstrated that the accuracy in T staging increased to 89.47% in EUS+MRI, a combination that is significantly more accurate compared with MRI examination. Compared with EUS, the difference was not statistically significant. In this study, due to T4 gastric cancer early micro-invasive outside the stomach, a patient was diagnosed as T3 stage using EUS and MRI, but T4 stage was confirmed by pathological staging subsequent to surgery. Two cases of depressed type early gastric cancer were accurately identified by EUS and treated with endoscopic mucosal resection, and followed up for five months without recurrence.

The sensitivity of EUS in lymph node is insufficient, particularly for D2 and D3 lymph node evaluation of EUS. This study suggested the accuracy of EUS in N0 stage detection was significantly higher than that of MRI, but significantly lower in the N2 and N3 stages. This may be caused by D2/3 lymph node stations beyond the depth of EUS probe detection. EUS can be employed for the evaluation of lymph node size, shape, borders, echo density and echo characteristics. Thus, the specificity of the lymph node metastasis is higher.

The evaluation of MRI in preoperative N staging is still in the exploratory stage. CT differential diagnosis of lymph node metastasis was used as the standard for MRI. Smaller lymph node identification remains a challenge in use of magnetic resonance image for diagnosis (10). MRI detection accuracy rate was 68.42% in this study, similar to previous results (11). Compared with EUS, MRI has certain advantages in gastric cancer staging for lymph node detection, particularly in D2/3 station detection. The disadvantages of MRI were that, besides the tumor infiltration, the pathological inflammation and fibrosis enhanced the signal, leading to a misdiagnosis.

EUS and MRI have a higher accuracy in gastric T and N staging. The combination of EUS and MRI can significantly improve the accuracy of preoperative T/N staging. Extremely early gastric cancer can be diagnosed by EUS, while endoscopic surgery has broad clinical application prospects. The EUS for N staging is insufficient, particularly for smaller, distant perigastric lymph nodes. However, EUS combined with MRI is expected to improve the accuracy for N staging.

## Figures and Tables

**Table I.** Accuracy of EUS, MRI and EUS+MRI in gastric T staging.

p Stages	EUS					MRI					EUS+MRI				
	T1	T2	T3	T4	Accuracy (%)	T1	T2	T3	T4	Accuracy (%)	T1	T2	T3	T4	Accuracy (%)
T1	9	1	0	0	90.00^c^	7	3	0	0	70.00	9	1	0	0	90.00^d^
T2	0	7	2	0	77.78^e^	0	6	3	0	66.67	0	9	1	0	90.00^f^
T3	0	2	13	0	86.67	0	2	11	2	73.33	0	2	13	0	86.67
T4	0	0	1	4	80.00	0	0	1	4	80.00	0	0	0	5	100.00
Total					86.84^a^					73.68					89.47^b^

EUS, endoscopic ultrasound; MRI, magnetic response imaging.

a EUS vs. MRI, P=0.04,

b MRI vs. EUS+MRI, P=0.035,

c EUS vs. MRI, P=0.032,

d EUS+MRI vs. MRI, P=0.032,

e EUS+MRI vs. EUS, P=0.04,

f EUS+MRI vs. MRI, P=0.002.

**Table II.** Accuracy of EUS, MRI and EUS+MRI in gastric N staging.

p Stages	EUS					MRI					EUS+MRI				
	N0	N1	N2	N3	Accuracy (%)	N0	N1	N2	N3	Accuracy (%)	N0	N1	N2	N3	Accuracy (%)
N0	15	0	0	0	100.00^a^	13	2	0	0	86.67	15	0	0	0	100.00^b^
N1	5	4	0	0	44.44	3	4	2	0	44.44	3	4	2	0	44.44
N2	2	5	5	0	45.45^c^	1	4	6	0	54.54	1	4	6	0	54.54^d^
N3	0	1	2	1	25.00	0	0	1	3	75.00	0	0	1	2	75.00
Total					65.78					68.42					71.05

EUS, endoscopic ultrasound; MRI, magnetic response imaging.

a EUS vs. MRI, P=0.032,

b MRI vs. EUS+MRI, P=0.032,

c EUS vs. MRI, P=0.021,

d EUS+MRI vs. MRI, P=0.021.
